# Longitudinal Biochemical and Behavioral Alterations in a Gyrencephalic Model of Blast-Related Mild Traumatic Brain Injury

**DOI:** 10.1089/neur.2024.0002

**Published:** 2024-03-14

**Authors:** Shiyu Tang, Su Xu, Donna Wilder, Alexandre E. Medina, Xin Li, Gary M. Fiskum, Li Jiang, Venkata R. Kakulavarapu, Joseph B. Long, Rao P. Gullapalli, Venkatasivasai Sujith Sajja

**Affiliations:** ^1^ Department of Diagnostic Radiology and Nuclear Medicine, Trauma, and Anesthesiology Research Center, University of Maryland School of Medicine, Baltimore, Maryland, USA; ^2^ Center for Advanced Imaging Research (CAIR), Trauma, and Anesthesiology Research Center, University of Maryland School of Medicine, Baltimore, Maryland, USA; ^3^ Blast Induced Neurotrauma Branch, Walter Reed Army Institute of Research, Silver Spring, Maryland, USA; ^4^ Department of Pediatrics, Trauma, and Anesthesiology Research Center, University of Maryland School of Medicine, Baltimore, Maryland, USA; ^5^ Department of Anesthesiology, Trauma, and Anesthesiology Research Center, University of Maryland School of Medicine, Baltimore, Maryland, USA; ^6^ Shock, Trauma, and Anesthesiology Research Center, University of Maryland School of Medicine, Baltimore, Maryland, USA

**Keywords:** biochemical and behavioral alterations, ferret, *in vivo* magnetic resonance spectroscopy, mild TBI, primary blast

## Abstract

Blast-related traumatic brain injury (bTBI) is a major cause of neurological disorders in the U.S. military that can adversely impact some civilian populations as well and can lead to lifelong deficits and diminished quality of life. Among these types of injuries, the long-term sequelae are poorly understood because of variability in intensity and number of the blast exposure, as well as the range of subsequent symptoms that can overlap with those resulting from other traumatic events (e.g., post-traumatic stress disorder). Despite the valuable insights that rodent models have provided, there is a growing interest in using injury models using species with neuroanatomical features that more closely resemble the human brain. With this purpose, we established a gyrencephalic model of blast injury in ferrets, which underwent blast exposure applying conditions that closely mimic those associated with primary blast injuries to warfighters. In this study, we evaluated brain biochemical, microstructural, and behavioral profiles after blast exposure using *in vivo* longitudinal magnetic resonance imaging, histology, and behavioral assessments. In ferrets subjected to blast, the following alterations were found: 1) heightened impulsivity in decision making associated with pre-frontal cortex/amygdalar axis dysfunction; 2) transiently increased glutamate levels that are consistent with earlier findings during subacute stages post-TBI and may be involved in concomitant behavioral deficits; 3) abnormally high brain *N*-acetylaspartate levels that potentially reveal disrupted lipid synthesis and/or energy metabolism; and 4) dysfunction of pre-frontal cortex/auditory cortex signaling cascades that may reflect similar perturbations underlying secondary psychiatric disorders observed in warfighters after blast exposure.

## Introduction

Neuropsychological symptoms in warfighters after exposures to blast have triggered considerable research interest in the pathophysiological manifestations of blast-induced traumatic brain injury (bTBI). Pre-clinical research models of blast are attractive tools to understand the pathogenesis of behavioral changes, identify relevant biomarkers, and characterize the neurobiological underpinnings of blast injury to potentially develop countermeasures to bTBI, including therapeutic interventions. Previously, we have established experimental conditions that yield ecologically valid blast-related brain injuries in rodents and closely resemble warfighters' exposures to primary blast as a foundation for understanding the injury pathogenesis.^1–4^ Despite the valuable insights these models have provided, there is a growing interest in identifying models of bTBI in other species with neuroanatomical features that more closely resemble the human brain.^5,6^ Unlike mice and rats, ferrets are gyrencephalical and bear a number of anatomical and physiological similarities to humans that, along with their size and related practical advantages, point to their great potential as an experimental model for high-throughput translational bTBI research that serves to develop countermeasures to the pathophysiological perturbations observed in warfighters exposed to blast.^5,7,8^

Neuroimaging plays a critical role in the acute setting to guide appropriate management based upon the detection of brain injuries that require intervention or further monitoring. Notably, in the setting of mild traumatic brain injury (mTBI) and bTBI, conventional computed tomography (CT) and structural magnetic resonance imaging (MRI) are most commonly used as diagnostic modalities to identify extent of injury in the brain parenchyma.^9–13^ In an attempt to better diagnose concussions and more subtle brain injuries, many additional advanced neuroimaging techniques are actively being pursued such as diffusion tensor imaging (DTI), which is more sensitive in detecting microstructural tissue damage after mTBI than conventional imaging tools.^14^ However, DTI has produced mixed findings in mild TBI patients,^15^ and a review showed an approximately equal number of studies reporting increased versus decreased fractional anisotropy (FA) in a wide range of white matter (WM) areas during the first 3 months after mTBI.^16^

Diffusion kurtosis imaging (DKI), which is a newer diffusion technique based on the non-Gaussian diffusion of water, is considered to better reflect diffusion in biological tissues, especially in brain areas with high tissue heterogeneity, such as gray matter.^17–19^ Hence, DKI has the potential to provide a distinct microstructural contrast in comparison with the DTI parameters. To date, few studies have investigated DKI in patients with mTBI^20–22^ and have focused primarily on the most common DKI metrics such as mean kurtosis (MK), axial kurtosis (AK), and radial kurtosis (RK). In these studies, lower MK and increased AK have been reported in WM and thalamus in patients with mTBI compared with healthy controls both in the acute, semiacute (within 1 month), and chronic (≥6 months) phases post-injury, and differences in DKI were demonstrated in the absence of DTI abnormalities. In line with this, DKI, but not DTI, changes were associated with hippocampal and cortical astrogliosis in one of our previous rat blast TBI studies.^23^ These studies support the view that DKI can be more sensitive to the pathology underlying mTBI than DTI.

Magnetic resonance spectroscopy (MRS) allows for *in vivo* measurement of biochemicals that are undetectable by conventional MRI, thereby holding potential to identify mTBI patients who could benefit from specific neuropsychiatric and cognitive rehabilitation.^24^ In the previous studies, changes observed in the brain with an *ex vivo* MRS were shown to correlate with pathophysiology and behavioral deficits after blast exposures.^25–28^ In our previous study using a rat model of direct cranial blast traumatic brain injury (TBI),^23^ we found delayed neurofunctional and pathological abnormalities subsequent to the brain injury that were silent on conventional T_2_-weighted imaging, but microstructural and metabolic changes were observed with DKI and proton MRS, respectively. Increased mean kurtosis, which peaked at 21 days post-injury, was observed in the hippocampus and internal capsule. Concomitant increases in myo-inositol (Ins) and taurine (Tau) were also observed in the hippocampus, whereas early changes at 1 day in glutamine (Gln) were observed in the internal capsule, all indicating glial abnormality in these regions.

In this study, to assess the subtle changes that are associated with blast exposure, we performed *in vivo* longitudinal DTI/DKI, MRS, and a behavioral test to assess the biochemical and behavioral profile alterations after blast exposure in a ferret model.

## Methods

### Ferret blast-related traumatic brain injury procedure

The experimental protocol was approved at the Walter Reed Army Institute of Research, and research was conducted in an Association for Assessment and Accreditation of Laboratory Animal Care (AAALAC) International-accredited facility in compliance with the Animal Welfare Act and all other federal statutes and regulations relating to animals and experiments involving animals, adhering to principles stated in the Guide for Care and Use of Laboratory Animals, NRC Publication 2011 edition, and the ARRIVE (Animal Research: Reporting of In Vivo Experiments) guidelines. In all, 18 male Fitch ferrets (1193 ± 19 g, age 108.1 ± 0.96 days) were subjected to bTBI using an advanced blast simulator that recreates “free-field”–like blast closely resembling that experienced by warfighters.^1,29^

Ferrets were anesthetized with 4% isoflurane for 8 min. An advanced blast simulator (ABS) was used for this study, which creates flow conditions much more closely resembling the Friedlander waves produced in the free field by improvised explosive devices and other explosive detonations, and was used to characterize exposures^29,30^ and eliminate artefactual blast wind, which has been the primary cause of substantial injury caused by exposures in constant diameter shock tubes. The ABS consisted of an 0.5-ft-long compression chamber separated from a 21-ft-long transition/expansion test section by rupturable VALMEX^®^ membranes (Mehler Texnologies, Inc., Martinsville, VA) or acetate membranes (Grafix Inc, Maple Heights, OH). The anesthetized ferret was secured in a longitudinal (i.e., head-on) orientation in the test section. Ferrets were exposed to a blast overpressure of ∼138 KPa with a 4- to 5-msec positive pressure duration. The critical biomechanical loading to the experimental subject was determined from both the static (Ps) and dynamic pressure (Pd) of the blast wave, which were fully recorded by a combination of side-on and head-on piezoresistive pressure gauges (Endevco, Depew, NY) using an Astro-med tmx-18 acquisition system at an 800,000-Hz sampling rate. This generated a model of blast TBI.

All sham animals in these experiments were subjected to isoflurane anesthesia, loading in the shock tube, and recovery procedures as described above, but were not exposed to the blast overpressure. Nine blast ferrets and 9 sham ferrets were included in this study.

### Imaging experiments

The imaging protocol was approved by the Institutional Animal Care and Use Committee (IACUC) of the University of Maryland, with IACUC No. 0519003. Imaging experiments were performed at 3 days, 7 days, 1 month, 3 months, and 6 months post-injury. *In vivo* magnetic resonance (MR) experiments were performed on a Bruker Biospec 7T scanner (Bruker BioSpin MRI GmbH, Ettlingen, Germany) run by Paravision 6.0. Anesthesia was maintained with around 3% isoflurane during the imaging session. A Bruker 30-mm single-turn surface coil was used as a receiver. A corresponding Bruker 86-mm-volume coil was used as a transmitter. An MR compatible small-animal monitoring and gating system (SA Instruments, Inc., Stony Brook, NY) was used to monitor animal respiration rate and body temperature, which was maintained at 37°C–38.5°C using a warm water bath circulation.

Coronal T_2_-weighted rapid acquisition with relaxation enhancement (RARE) sequences was used to identify key neuropathologies such as bleeding, hematomas, edema, or gross changes in brain structure. The imaging parameters were: repetition time (TR), 3149 msec; echo time (TE_eff1/_TE_eff2_), 22/66 msec; field of view, 4.5 × 4.5 cm^2^; RARE factor, 2; matrix, 256 × 256; averages, 2; slice thickness, 1.5 mm; and image slices, 20.

DTI and DKI data were collected using an echo planar imaging spin echo diffusion scheme with a total of 30 diffusion directions in the coronal view. In total, five b = 0 images were collected with: TE, 24.58 ms; TR, 2500 ms; repetitions, 2; and matrix size, 128 × 128. The rest of the parameters matched to the corresponding T_2_-weighed images. A scan with the b-value set to 1000 and 2000 s/mm^2^ was collected. The diffusion gradient separation was 9.34 ms. The diffusion gradient duration was 3.0 ms.

**FIG. 1.**
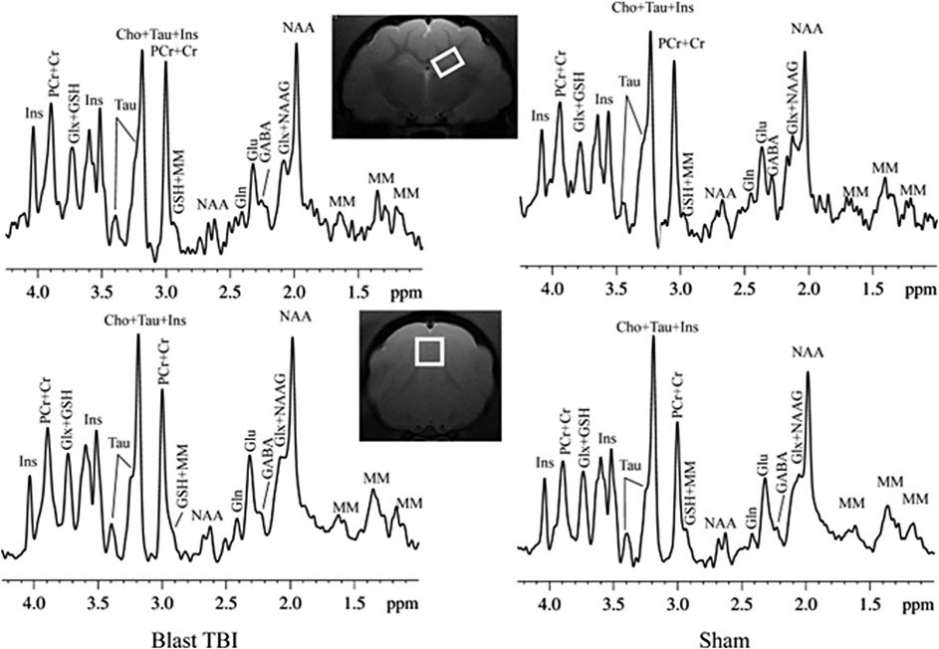
Demonstration of the MRS voxel in hippocampal (**A**) and pre-frontal cortex (**B**) and spectrums of a blast and sham ferret at 3 days post-blast. NAA, *N*-acetyl-aspartate; Glu, glutamate; Gln, glutamine; GABA, gamma-aminobutyric acid; GSH, glutathione; PCr, phosphocreatine; Cr, creatine; Cho, choline; Ins, myo-inositol; Tau, taurine; Glx, glutamate/glutamine complex; NAAG, N-acetylaspartylglutamate; MM, macro molecules; MRS, magnetic resonance spectroscopy.

^1^H-MRS data were obtained from a voxel (3.5 × 3.5 × 3.5 mm^3^) that covered the mPFC and left hippocampus (3.5 × 2 × 4 mm^3^; Fig. 1). A short-TE point-resolved spectroscopy pulse sequence (TR/TE = 2500/10 ms, numerical aperture = 360) was used for MRS data acquisition.^31^ The unsuppressed water signal from the prescribed voxel was used as a reference for determining the specific metabolite concentrations.

### Behavioral assessment

**FIG. 2.**
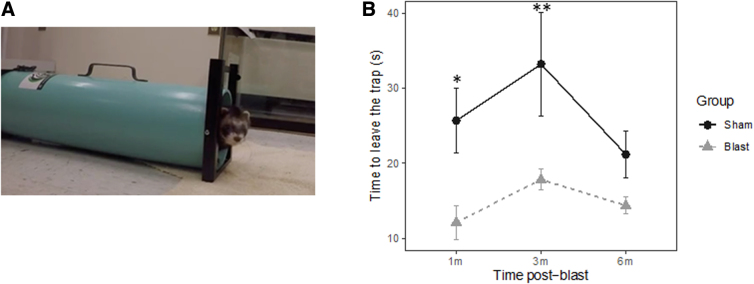
(**A**) In-house trap test for the ferret. (**B**) Time required to walk out from the trap of the sham and blast-exposed ferrets at 1, 3, and 6 months post-blast. *p < 0.05, **p < 0.01, differences between sham and blast animals.

Behavioral assessments were conducted at 1, 3, and 6 months post-injury. The primary behavioral assessment performed in this study was a trap test for ferrets that was developed in-house. The test utilized a modified skunk trap with a panel to control the opening and closing of the trap at one end (Fig. 2A). The ferret was put inside the trap and allowed to acclimate for 2 min. After the acclimation, the panel was opened and the time taken to fully exit the trap (from nose at the opening to rear feet out) was recorded. The test was designed to measure level of anxiety and impulsivity.

### Histology

At 6 months post-blast, after behavior and imaging studies, animals were deeply anesthetized and perfusion-fixed using 4% paraformaldehyde at the University of Maryland. Fixed brains were sent to FDNeuroTechnologies Inc. (Columbia, MD) for further processing for silver staining. Briefly, paraffin-embedded sections were cut to 50 μm and stained with the FD NeuroSilver™ Kit II in accordance with the manufacturer's protocol. Researchers were blinded to exposure groups and used light microscopy for imaging of brain regions of interest (ROIs). ImagePro 9 software (Media Cybernetics, Rockville, MD) was used to assess the silver-staining–positive regions.

### Image analysis

**FIG. 3.**
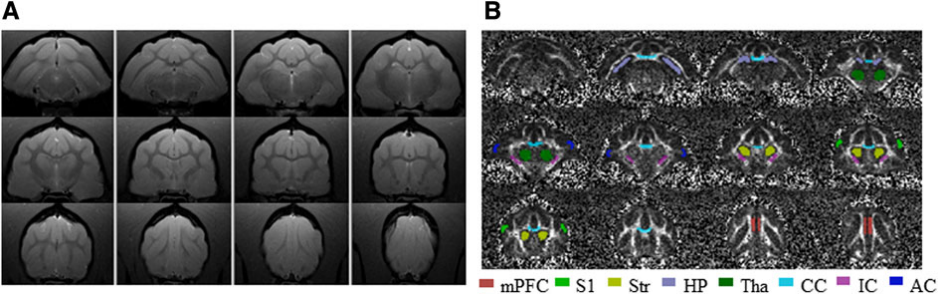
(**A**) Ferret anatomical MRI. (**B**) ROIs for DKI and DTI parameters extraction from FA maps. mPFC, medial pre-frontal cortex; S1, primary somatosensory cortex; Str, striatum; HP, hippocampus; Tha, thalamus; CC, corpus callosum; IC, internal capsule; AC, auditory cortex; MRI, magnetic resonance imaging; ROIs, regions of interest; DKI, diffusion kurtosis imaging; DTI, diffusion tensor imaging.

DKI and DTI model fitting was performed using customized diffusion kurtosis software,^32^ and parametric maps were calculated for mean diffusivity (MD), FA, axial diffusivity (AD), radial diffusivity (RD), MK, AK, and RK. The ROIs, including medial pre-frontal cortex (mPFC), primary somatosensory cortex, striatum, hippocampus, thalamus, corpus callosum, internal capsule, and auditory cortex, were manually defined on the FA images while using the T_2_-weighted image for anatomical reference in FSLeyes (Fig. 3). Values of MD, FA, AD, RD, MK, AK, and RK were extracted respectively from each generated map using the manually defined ROIs. The detailed procedure has been published previously.^31^

^1^H-MRS data were fitted using the LCModel package (version 6.3-0G; LCModel Inc., Oakville, ON, Canada).^33^ A threshold of the Cramer-Rao lower bounds ≤35 % was used to select metabolite concentrations that had an acceptable level of quantification reliability.^32^

### Statistical analysis

Statistical analysis was performed in R (version 4.0.2; R Core Team, 2013). Repeated-measures analysis of variance (ANOVA) was performed on imaging and behavioral measures with group as between-subject effect and time as within-subject effect. Interaction effect between group and time was also considered. *Post hoc* pairwise comparison was performed with Tukey's method when significant main effects or interaction were detected.

## Results

### Imaging experiments

T_2_-weighted MRI did not indicate the presence of hematoma, hemorrhage, or edema after bTBI in any of our experimental subjects (data not shown) at 3 days to 6 months post-exposure. However, we detected brain tumors in 2 sham ferrets. One ferret showed a benign tumor located in the cerebellum at the first MRI time point, which did not significantly grow or change shape during the total 6 months. Because the location of the tumor was not included in our ROIs and the experimental subject did not show any abnormal behavior or weight gain compared to its peers, we kept this ferret in our study. Another sham ferret showed a benign tumor located in the left hippocampal region at the first time point and was excluded from this study because the region was included in our ROIs. Therefore, the ferrets used in the sham group decreased from 9 to 8 over the course of the study.

No microstructural disruption of the TBI was statistically distinguished by the DTI and DKI data evaluated from the brain regions selected in this cohort. However, a widespread effect of time was shown on diffusion parameters across the brain, which reflected the ferret brain structural maturation (from approximately 3.5 to 9.5 months; Figure 3 and Tables 1 and 2). During the 6 months, globally increased FA, MK, RK, and AK values and decreased MD, AD, and RD values were observed in the ferrets.

**Table 1.** Effect of Time on DTI/DKI Parameters

Visit	MD		AD		RD		FA		MK		AK		RK	
	F	p	F	p	F	p	F	p	F	p	F	p	F	p
mPFC	3.08	0.022(d)	4.55	0.003(d)			2.80	0.034(i)	10.28	<0.001(i)	21.85	<0.001(i)		
S1	3.57	0.011(d)			7.74	<0.001(d)	7.81	<0.001(i)	15.51	<0.001(i)	6.18	<0.001(i)	7.11	<0.001(i)
Str	2.78	0.026(d)	9.4	<0.001(d)	5.91	<0.001(d)			6.06	<0.001(i)			6.8	<0.001(i)
HP					4.86	0.002(d)	3.9	0.006(i)	4.57	0.003(i)			2.94	0.026(i)
Tha					3.42	0.009(d)	4.12	0.005(i)	6.76	<0.001(i)			6.64	<0.001(i)
CC	8.08	<0.001(d)	6.86	<0.001(d)	4.32	0.004(d)			29.12	<0.001(i)	16.57	<0.001(i)	4.65	0.002(i)
IC			3.78	0.008(d)	6.93	<0.001(d)	7.7	<0.001(i)	3.44	0.013(i)			3.97	0.006(i)

mPFC, medial pre-frontal cortex; S1, primary somatosensory cortex; Str, striatum; HP, hippocampus; Tha, thalamus; CC, corpus callosum; IC, internal capsule; MD, mean diffusivity; FA, fractional anisotropy; AD, axial diffusivity; RD, radial diffusivity; MK, mean kurtosis; AK, axial kurtosis; RK, radial kurtosis; I, increased; d, decreased.

**Table 2.** Widespread Effect of Time Was Shown on Diffusion Parameters Across the Brain

MK	3d vs. 1m	3d vs. 3m	3d vs. 6m	7d vs. 1m	7d vs. 3m	7d vs. 6m	1m vs. 6m	3m vs. 6m
mPFC		*p* = 0.008	*p* = 0.006	*p* = 0.006	*p* < 0.001	*p* < 0.001		
S1	*p* = 0.038	*p* = 0.001	*p* < 0.001		*p* = 0.005	*p* < 0.001	*p* = 0.002	
Str			*p* = 0.011		*p* = 0.013	*p* = 0.001		
HP					*p* = 0.04	*p* = 0.001		
Tha			*p* = 0.012		*p* = 0.016	*p* < 0.001	*p* = 0.007	
CC	*p* < 0.001	*p* < 0.001	*p* < 0.001	*p* = 0.018	*p* < 0.001	*p* < 0.001	*p* < 0.001	*p* = 0.01
IC			*p* = 0.005					

**AK**	**3d vs. 7d**	**3d vs. 3m**	**3d vs. 6m**	**7d vs. 1m**	**7d vs. 3m**	**7d vs. 6m**	**1m vs. 3m**	**1m vs. 6m**
mPFC	*p* = 0.021	*p* = 0.006	*p* < 0.001	*p* < 0.001	*p* < 0.001	*p* < 0.001		*p* = 0.002
S1			*p* = 0.007		*p* = 0.014	*p* = 0.001		
CC		*p* = 0.012	*p* < 0.001		*p* < 0.001	*p* < 0.001	*p* = 0.019	*p* < 0.001

**RK**	**3d vs. 1m**	**3d vs. 3m**	**3d vs. 6m**	**7d vs. 3m**	**7d vs. 6m**	**1m vs. 3m**	**1m vs. 6m**	
S1		*p* = 0.017	*p* < 0.001		*p* = 0.004			
Str			*p* = 0.001		*p* < 0.001			
HP					*p* = 0.038			
Tha				*p* = 0.002	*p* = 0.001	*p* = 0.048	*p* = 0.038	
CC	*p* = 0.042		*p* = 0.001					
IC	*p* = 0.016		*p* = 0.007					

**MD**	**3d vs. 6m**	**7d vs. 6m**	**1m vs. 6m**					
mPFC		*p* = 0.047						
S1	*p* = 0.011							
Str	*p* = 0.04							
CC	*p* < 0.001	*p* < 0.001	*p* = 0.004					

**AD**	**3d vs. 3m**	**3d vs. 6m**	**7d vs. 6m**	**1m vs. 6m**				
mPFC		*p* = 0.002	*p* = 0.01					
CC			*p* < 0.001	*p* = 0.001				
IC	*p* = 0.005	*p* = 0.043						

**RD**	**3d vs. 1m**	**3d vs. 3m**	**3d vs. 6m**	**7d vs. 6m**				
S1		*p* = 0.006	*p* < 0.001	*p* = 0.002				
Str			*p* < 0.001	*p* = 0.008				
HP			*p* = 0.009	*p* = 0.005				
Tha				*p* = 0.012				
CC			*p* = 0.003					
IC	*p* = 0.003	*p* = 0.028	*p* < 0.001					

**FA**	**3d vs. 1m**	**3d vs. 3m**	**3d vs. 6m**	**7d vs. 3m**				
S1		*p* = 0.001	*p* < 0.001	*p* = 0.015				
Str	*p* = 0.037	*p* < 0.001	*p* < 0.001					
HP								
Tha				*p* = 0.042				
IC	*p* = 0.004	*p* = 0.001	*p* < 0.001					

mPFC, medial pre-frontal cortex; S1, primary somatosensory cortex; Str, striatum; HP, hippocampus; Tha, thalamus; CC, corpus callosum; IC, internal capsule; MD, mean diffusivity; FA, fractional anisotropy; AD, axial diffusivity; RD, radial diffusivity; MK, mean kurtosis; AK, axial kurtosis; RK, radial kurtosis; i, increased; d, decreased.

MRS data showed a significant group main effect for glutamate (Glu; *p* = 0.047) and *N*-acetyl-aspartate (NAA; *p* = 0.048) in the mPFC. Both metabolites increased significantly in the blast ferrets. Glu increased transiently at 1 month post-blast (*p* = 0.024), whereas NAA remained high at both 1 and 3 months post-blast (*p* = 0.039 and *p* = 0.047, respectively) in the blast ferrets.

**FIG. 4.**
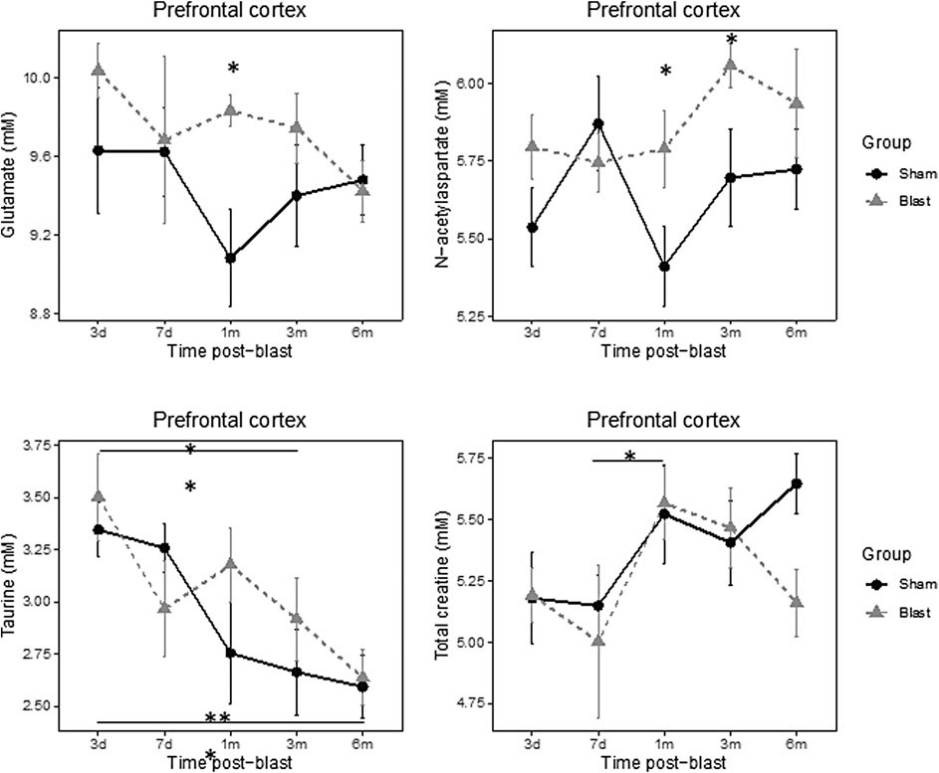
Effect of blast on cerebral metabolomics profiles in pre-frontal cortex. *p < 0.05, **p < 0.01, between group or time differences.

**FIG. 5.**
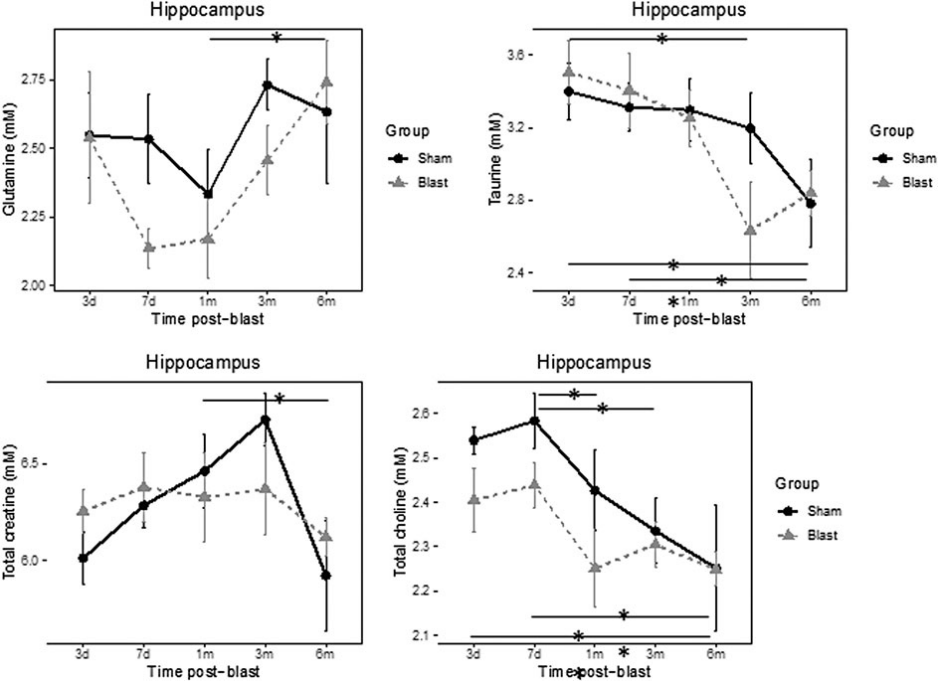
Effect of blast on cerebral metabolomics profiles in hippocampus. *p < 0.05, **p < 0.01, between group or time differences.

In addition, a significant effect of time was observed on Tau (*p* < 0.001) and total creatine (tCr; *p* = 0.026), which reflected developmental changes. Tau was significantly lower at 3 and 6 months when comparing to 3 days post-blast (*p* = 0.006 and *p* < 0.001, respectively) in both groups. tCr significantly increased in both groups from 7 days to 1 month post-blast (*p* = 0.028; Fig. 4). In the hippocampus, a significant effect of time was observed with Gln (*p* = 0.024), Tau (*p* = 0.003), tCr (*p* = 0.018), and total choline (tCho; *p* < 0.001; Fig. 5). Gln increased significantly from 1 to 6 months post-blast (*p* = 0.031). Tau decreased over time and was significantly lower at 3 months compared to 3 days post-blast (*p* = 0.035) and was also significantly lower at 6 months compared to both 3- (*p* = 0.009) and 7-day (*p* = 0.04) measurements post-blast. tCr was significantly lower at 6 months compared to 3 months post-blast (*p* = 0.02). tCho significantly decreased from 3 days to 6 months post-blast (3 days vs. 6 months, *p* = 0.007; 7 days vs. 1 month, *p* = 0.03; 7 days vs. 3 months, *p* = 0.014; 7 days vs. 6 months, *p* = 0.001).

### Behavior assessment

A significant main effect of group was detected for the time animals spent in the trap (*p* = 0.01). The blast ferrets spent significantly less time to exit the trap than the sham ferrets at 1 and 3 months post-blast (*p* = 0.012 and *p* = 0.005, respectively). The difference did not reach statistical significance at 6 months post-blast (*p* = 0.256; Fig. 2B).

### Histology

**FIG. 6.**
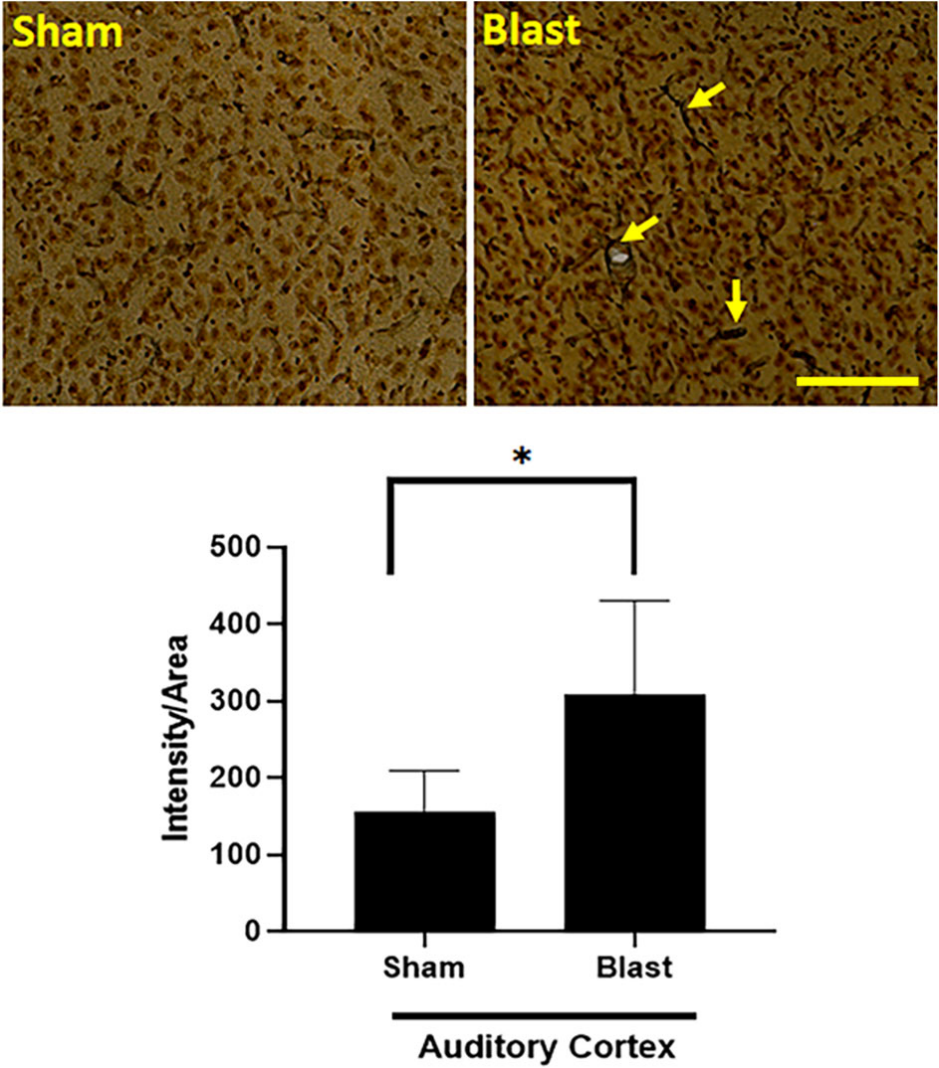
Effect of blast on argyrophilic inclusions in the auditory cortex. Note a significant increase in the density of silver-stained inclusions at 6 months post-blast. *p < 0.05. Scale bar = 100 μm.

Silver staining showed a significant increase in argyrophilic inclusions in the anterior ectosylvian gyri area of the auditory cortex (*p* < 0.05; Fig. 6). No statistically significant changes were observed in the thalamus and hippocampal regions examined at 6 months after blast exposure (data not shown).

## Discussion

We report here that blast injury in the gyrencephalic ferret resulted in increased behavioral impulsivity, a transient rise in Glu and abnormal elevations of NAA in the pre-frontal cortex, and argyrophilic inclusions in auditory cortex. In addition, the ferrets used in this study showed decreased levels of Tau and an increase of tCr over time. Taken together with previous pre-clinical and clinical studies, these findings associated with neuropathological and -behavioral consequences of blast exposure have similarities to clinical findings after blast exposures, where subtle long-term changes have been noted after an mTBI.^33^

In the trap test, the blast-exposed ferrets spent less time observing the surrounding environment and exited the opening faster than the sham ferrets, which likely represents a possible increased impulsive decision making, which has been earlier attributed to a dysfunction in the pre-frontal cortical/amygdalar axis post-blast.^34^ Previous research has established a link between decreased impulse control and pre-frontal alterations in patients with TBI.^34–36^ Impulsive behavior can either increase the chance of experiencing TBI, or TBI can lead to a decline in impulse control. It is therefore likely that the present observation of increased impulsivity may be attributable to the effect of blast.^37^

Our findings of increased Glu are consistent with earlier reports that showed excitotoxicity and associated cognitive dysfunctions during acute and subacute stages post-TBI,^40^ which were also observed in a rat model of blast exposure. An enhanced level of Glu in the pre-frontal cortex has also been observed in schizophrenia, bipolar disorder, and major depression.^38–40^ Considering that TBI is a risk factor for schizophrenia^41^ and psychotic disorders,^42^ our MRS results, together with the observed increased impulsivity, suggest a link between dysfunction of the pre-frontal cortex and potential secondary psychiatric disorders after mild blast TBI.

NAA plays an important role in lipid synthesis and energy metabolism. Abnormally high brain NAA levels correlated with deficient axonal myelin sheath development.^43^ The NAA in TBI ferrets showed significantly higher levels compared to sham animals at both 1 and 3 months, which may indicate a disruption of the utilization of NAA in lipid synthesis and/or energy metabolism at the time. Tau was observed to decrease with time in both groups, in agreement with previous reports that *in vivo* ^1^H MRS decreases in rodents and humans during maturation.^44–47^ In the perinatal period, a sufficient level of taurine is vital to proper brain development.^48–51^ The increase in tCr with time also reflects a maturation change, which is supported by previous reports.^44–47^ The continuation of trends in changes of Tau and tCr, which start in the fetal stage of development, has been postulated to reflect the increase in neural activity associated with maturation.^52^

Water diffusion patterns, which are measured by DTI and DKI, have been considered useful reflections of the underlying axonal organization of the brain.^53^ In this study, blast overpressure did not change the diffusion parameters, which diverges from findings in previous studies on mTBI in rats and humans.^20–22^ This discrepancy may likely be attributable to the mild nature of the bTBI model evaluated in this study. This study instead revealed maturation changes of DKI parameters in a group of brain regions. Studies in humans have shown that the maturation of WM involves a phase of axonal myelination, which corresponds to the ensheathment of oligodendroglial processes around the axons.^19,54,55^ Similar to our findings, clinical studies have shown an increase FA values in blast-exposed veterans when compared to non-blast cohorts, suggesting a possible clinical relevance of this ferret injury model. As has been shown in both pre-clinical and clinical studies, the increase in the FA values corresponds with astrogliosis after blast exposure.^7,8,56^

The argyrophilic inclusions in the auditory cortex of the blast-exposed ferret likely reflects ongoing neurodegeneration and axonal degeneration, even at 6 months post-blast, which is noteworthy given that the auditory system is highly sensitive to blast injury. The absence of argyophilic inclusions in the other regions examined, including the hippocampus and thalamus, are possibly reflective of earlier degeneration that did not persist through 6 months post-blast. However, it is possible that brain regions other than those examined in the present study (auditory cortex, hippocampus, and thalamus) might display increased intensity of argyrophilic inclusions.

Collectively, these findings show potential maturation changes at a microscopic level, which are apparent with advanced imaging techniques such as DKI and MRS, while also demonstrating the clinical relevance of the readouts that a ferret model can present. Using the ferret as a gyrencephalic animal model to evaluate the effects of blast injuries shows that they display several features resembling those observed in human victims of blast exposures. In particular, the ferret blast TBI model showed a disrupted functioning of the pre-frontal cortex, which could possibly link to the psychiatric disorders associated with blast exposure. As these and other corroborations with clinical findings emerge, we anticipate that they will show ferrets to be a highly translatable model to understand blast-related TBI and potentially point to their utility for future therapeutic evaluations, which, stemming from greater neuroanatomical similarities, may have improved translatability than has been achieved with rodent injury models.

## Transparency, Rigor, and Reproducibility Summary

This study used 18 male Fitch ferrets (3 months old; weight ∼1100 g). Sample size was 9 ferrets per group; that is, sham controls (*n* = 9) and blast exposure group (*n* = 9), based on the power analysis calculator (www.statisticalsolutions.net) with input values of anticipated effect 27% with 30% standard deviation at 95% confident intervals along with an alpha (0.05) and power of the test (0.8). Investigators who performed MRI, ^1^H-MRS, and histopathological analysis were blinded to groups. Statistical analysis was performed in R (version 4.0.2; R Core Team, 2013). Repeated-measures ANOVA was performed on imaging and behavioral measures with group as between-subject effect and time as within-subject effect. Interaction effect between group and time was also considered. *Post hoc* pairwise comparison was performed with Tukey's method when significant main effects or interaction were detected.

## Abbreviations Used

ABS: advanced blast simulator
AD: axial diffusivity
AK: axial kurtosis
ANOVA: analysis of variance
bTBI: blast-induced traumatic brain injury
CT: computed tomography
DKI: diffusion kurtosis imaging
DTI: diffusion tensor imaging
FA: fractional anisotropy
Gln: glutamine
Glu: glutamate
IACUC: Institutional Animal Care and Use Committee
Ins: myo-inositol
MD: mean diffusivity
MK: mean kurtosis
mPFC: medial pre-frontal cortex
MR: magnetic resonance
MRI: magnetic resonance imaging
MRS: magnetic resonance spectroscopy
mTBI: mild traumatic brain injury
NAA: *N*-acetyl-aspartate
RARE: rapid acquisition with relaxation enhancement
RD: radial diffusivity
RK: radial kurtosis
ROIs: regions of interest
Tau: taurine
TBI: traumatic brain injury
tCho: total choline
tCR: total creatine
TE: echo time
TR: repetition time
WM: white matter
